# Modeling of Body Weight Metrics for Effective and Cost-Efficient Conventional Factor VIII Dosing in Hemophilia A Prophylaxis

**DOI:** 10.3390/pharmaceutics9040047

**Published:** 2017-10-17

**Authors:** Alanna McEneny-King, Pierre Chelle, Severine Henrard, Cedric Hermans, Alfonso Iorio, Andrea N. Edginton

**Affiliations:** 1 School of Pharmacy, University of Waterloo, Waterloo, ON N2L 3G1, Canada; amceneny@uwaterloo.ca (A.M.-K.); pierre.chelle@uwaterloo.ca (P.C.); 2 Louvain Drug Research Institute, Clinical Pharmacy Research Group and Institute of Health and Society (IRSS), Université catholique de Louvain, 1348 Brussels, Belgium; severine.henrard@uclouvain.be; 3 Haemostasis and Thrombosis Unit, Division of Haematology, Cliniques universitaires Saint-Luc, Université catholique de Louvain, 1348 Brussels, Belgium; cedric.hermans@uclouvain.be; 4 Department of Health Evidence, Research Methods and Impact, McMaster University, Hamilton, ON L8S 4L8, Canada; iorioa@mcmaster.ca; 5 Department of Medicine, McMaster University, Hamilton, ON L8S 4L8, Canada

**Keywords:** hemophilia A, conventional factor VIII, dose metrics, obesity, population pharmacokinetics

## Abstract

The total body weight-based dosing strategy currently used in the prophylactic treatment of hemophilia A may not be appropriate for all populations. The assumptions that guide weight-based dosing are not valid in overweight and obese populations, resulting in overdosing and ineffective resource utilization. We explored different weight metrics including lean body weight, ideal body weight, and adjusted body weight to determine an alternative dosing strategy that is both safe and resource-efficient in normal and overweight/obese adult patients. Using a validated population pharmacokinetic model, we simulated a variety of dosing regimens using different doses, weight metrics, and frequencies; we also investigated the implications of assuming various levels of endogenous factor production. Ideal body weight performed the best across all of the regimens explored, maintaining safety while moderating resource consumption for overweight and obese patients.

## 1. Introduction

Hemophilia A is an inherited bleeding disorder resulting from a deficiency in clotting factor VIII (FVIII), causing spontaneous and recurring joint bleeds, eventually leading to arthropathy and premature death if left untreated. The mainstay of severe hemophilia treatment is prophylactic replacement of the missing factor. The typical aim of prophylaxis is to maintain a clotting factor level of at least 1 IU dL^−1^, based on the observation that patients with moderate hemophilia (i.e., those with baseline factor levels >1 IU dL^−1^) are less prone to the spontaneous bleeds and subsequent arthropathy seen in more severe cases [1]. In a study of 65 boys with severe hemophilia A, only regular prophylactic infusions were shown to prevent joint damage as compared to on-demand treatment [2]. While there is global unanimity that prophylaxis should be initiated before joint disease is sustained [3,4], the implementation of this approach is quite variable [5]. No optimal dosing regimen has been identified; instead, an individualized approach that accounts for the patient’s physical activity, current (and accepted future) musculoskeletal condition, and the availability of resources has been suggested [6,7]. Ideally, the patient’s pharmacokinetic (PK) profile is taken into account to define a truly individualized regimen that optimizes both safety and resource utilization [8]. To facilitate the adoption of PK-based dosing regimens, tools such as the Web Accessible Population Pharmacokinetics Service—Hemophilia (WAPPS-Hemo [9,10]) provide estimates of individual PK parameters from a minimal number of samples by leveraging population PK data. Despite the development of these platforms, the majority of hemophilia patients are still dosed according to total body weight, as initially proposed by Ingram in 1981 [11]. For instance, hemophilic children in Canada are started on a once-weekly regimen (50 IU kg^−1^), then step up to either twice weekly (30 IU kg^−1^) or every 48 h (25 IU kg^−1^) as required; prophylaxis regimens in the Netherlands (Utrecht protocol: 15–30 IU kg^−1^ three times per week) and Sweden (Malmö protocol: 25–40 IU kg^−1^ three times per week), though proposing different intensities and targeting different levels, are based on the same principle [12].

The normalization of life expectancy of individuals with hemophilia brings new challenges to hemophilia care. Overweight and obesity rates amongst hemophiliacs now match the epidemic proportions that are seen in the general population [13]. A 2011 study conducted in Ontario found 28.8% of enrolled hemophiliacs were overweight or obese, compared to 26% of healthy controls [14]. Obesity also comes with a higher risk for hemophilic arthropathy; joint range of motion has been shown to negatively correlate with body mass index (BMI) [15]. Furthermore, the total body weight-based dosing regimen currently used in hemophilia treatment may not be appropriate for overweight and obese populations. Calculations for weight-adjusted dosing are based on the following formula:

(1) $$\mathrm{Dose}\;(\mathrm{IU})=\frac{\mathrm{total}\;\mathrm{body}\;\mathrm{weight}\;\left(\mathrm{kg}\right)\times \mathrm{desired}\;\mathrm{increase}\;\mathrm{in}\;\mathrm{FVIII}\;\mathrm{level}\;(\%)}{\mathrm{IVR}}$$

In vivo recovery (IVR) is a parameter used to describe clotting factor pharmacokinetics, and reflects the rise in factor activity (in this case, FVIII) after a dose is administered. Although it has been suggested that an individual IVR value be determined for each patient [16], typically an IVR of 2 IU dL^−1^/IU kg^−1^ is assumed. For example, a desired increase to normal FVIII levels (100%) would lead to a 50 IU kg^−1^ dose being administered. However, the assumption that IVR equals 2 for all is not always valid. A study by Henrard et al. found that overweight patients (BMI > 29.6 kg·m^−2^) had a median IVR of 2.70, while underweight patients (BMI < 20.3 kg·m^−2^) had a median IVR of 1.60 [17].

The emerging proportion of overweight and obesity in the general population has prompted research efforts aimed at identifying pharmacokinetic differences (and the corresponding dose adjustments) in this population. The relationship between body size and clearance is well established; a 2012 systematic review of this topic found that more than half of all identified models for clearance included a covariate for body size, most commonly as a power function [18]. Obesity specifically influences several factors affecting drug disposition, including body composition, metabolism by CYP450 enzymes, and plasma protein levels [19]. The most striking differences are observed for highly lipophilic drugs, where volume of distribution changes dramatically in the obese population [20]. However, this is not the case for clotting factor concentrates. FVIII concentrates are typically confined to the vascular space, with volumes of distribution approximating plasma volume (48 mL·kg^−1^) [21]. Since vasculature represents a very small fraction (0.005–0.010) of adipose tissue volume [22], an excess (or scarcity) of fat does not significantly alter the volume of distribution of FVIII. As a result, overweight and obese patients are likely overdosed when dose is calculated using total body weight [23]. A similar issue has been noted for dosing of unfractionated heparin, another compound whose volume of distribution is approximately equal to the plasma volume; obese children achieved comparable anticoagulation at a lower weight-based dose [24]. Hemophilia treatment is expensive, with annual costs in the hundreds of thousands for those on prophylaxis [2], and while prophylaxis does achieve better health outcomes, these come at a significant cost that is not automatically offset by prevention of other expenses [25]. As the clotting factor itself represents the majority of the cost of prophylaxis [26], overdosing can introduce a significant waste of resources [27]. This study will explore alternative dosing regimens that optimize both safety and resource utilization in overweight and obese hemophiliacs.

## 2. Methods

Population generation, simulation, and data analysis were all conducted in Matlab R2009.

### 2.1. Population Generation

The generated population of virtual individuals consists of two equal sized bins classified by BMI using the cut-offs defined by Henrard et al. [17] The first group consists of average weight subjects (BMI between 20.3 and 29.6 kg·m^−2^); the second group represents an overweight and obese population with BMI between 29.6 kg·m^−2^ and 40.0 kg·m^−2^. These cut-off values for BMI were found to be the strongest predictors of FVIII IVR. Each group contains 1000 simulated subjects with a uniform BMI distribution. Heights were derived from the distribution provided by the NHANES database [28]. A uniform distribution of BMI’s was simulated and the total body weights were calculated as the product of BMI and the square of height.

### 2.2. Definitions of Weight Metrics

The following weight metrics were defined for each virtual patient from their simulated total body weight (TBW, kg), height (HT, cm) and BMI (kg·m^−2^):

1. Lean body weight (LBW) [29]
  (2) $$\mathrm{LBW}=\frac{9270\times \mathrm{TBW}}{6680+216\times \mathrm{BMI}},$$
2. Ideal body weight (IBW—Lorentz formula)
  (3) $$\mathrm{IBW}=\mathrm{HT}-100-\left(\frac{\mathrm{HT}-150}{4}\right),$$
3. Adjusted body weight (ABW)
  (4) $${\mathrm{ABW}}_{25}=\mathrm{IBW}+0.25\times (\mathrm{TBW}-\mathrm{BW}),$$
  (5) $${\mathrm{ABW}}_{40}=\mathrm{IBW}+0.4\times (\mathrm{TBW}-\mathrm{IBW}).$$

We used the semi-mechanistic model for LBW developed by Janmahasatian et al. [30] as it has been found to better describe the full range of adult heights and weights [20]. IBW was calculated using Lorentz’s formula, which takes into account the patient’s height and sex but not total body weight. ABW was the first weight metric intended for use in pharmacokinetic studies; it involves adding a proportion of the excess weight above IBW [30]. This proportion is variable, ranging from 25–50%, with 40% being used most commonly; in this study, we examined both 25% (ABW_25_) and 40% (ABW_40_) correction factors. Correlation plots for all body size metrics are presented in Supplementary Figures S1 and S2 for normal and overweight/obese individuals, respectively.

### 2.3. Population Pharmacokinetic Model

Simulations were performed using the 2-compartment structure described by Garmann et al. [31] for BAY 81-8973 (Kovaltry^®^, Bayer, Leverkusen, Germany), built on 183 subjects. Of the 109 patients above 18 years of age, the BMI range was 15.0–38.3 kg∙m^−2^. The details of the model structure are presented in Table 1. For each simulated individual, PK parameters were calculated. Each virtual individual was then dosed based on various weight metrics and their PK was simulated.

### 2.4. Simulation and Assessment of Treatment Regimens

For each virtual individual, FVIII levels and individual PK parameters were simulated assuming a baseline factor level of 0.5 IU dL^−1^. FVIII levels were simulated using time steps of 0.2 h following dosing regimens for four weeks to ensure that steady state was reached, and results from the 5th week were used in subsequent analysis steps. In a first instance, we analyzed a typical dosing strategy (20 IU kg^−1^ TBW every 48 h) to evaluate its appropriateness.

We then simulated various regimens wherein equal doses were given at regular intervals (i.e., 48 h). Each patient was dosed from 10 IU kg^−1^ for each weight metric (10 IU kg^−1^ of TBW, 10 IU kg^−1^ of LBW, etc.) up to 210 IU kg^−1^. Initially, the dose step was 2 IU kg^−1^ for doses up to 100 IU kg^−1^ and 10 IU kg^−1^ for doses between 100 and 210 IU kg^−1^. After reviewing the results, the dose step was reduced to 0.1 IU kg^−1^ between 20 and 30 IU kg^−1^, as this was the range of most interest. A regimen was considered to be safe for a BMI group if 95% of the simulated population within that group had factor levels above 1 IU dL^−1^ at all times (C_min_ ≥ 1 IU dL^−1^). The lowest dose per weight metric that met this safety criterion was identified and considered to be the optimal regimen for that particular metric and BMI group. A secondary measure of safety was the 95th quantile for time spent below 1 IU dL^−1^; in other words, the amount of time per week spent below trough for the 5% of the population not meeting the safety criteria. To evaluate economic differences between regimens, we calculated the mean weekly consumption on each optimal regimen to determine which dosing regimen met safety requirements while minimizing resource expenditure. This process was then repeated for a Monday-Wednesday-Friday (M-W-F) dosing schedule. For these simulations, the optimal dose for each metric (determined in the previous simulations) was administered on Monday and Wednesday, and the Friday dose was increased until the safety criterion was reached. To evaluate the importance of the earlier assumption of 0.5 IU dL^−1^ baseline, we repeated the above simulations assuming a baseline of 0 IU dL^−1^ to observe if similar trends emerged.

## 3. Results

Simulations of the typical regimen of 20 IU kg^−1^ TBW every 48 h were completed and the results are summarized in Table 2. We then investigated the hypothesis that a TBW-based dosing regimen results in overdosing in overweight and obese patients by determining the TBW-based dose required to meet the 1 IU dL^−1^ safety criterion in 95% of these patients. At a dose of 20 IU kg^−1^ TBW, the median minimum concentration (C_min_) throughout the week for these patients was 5.4 IU dL^−1^; the average consumption associated with this dosing regimen was 7.25 × 10^3^ IU per person per week. However, this population requires only 14 IU kg^−1^ TBW to meet the 95% safety criterion, which corresponds to an average weekly consumption of 5.07 × 10^3^ IU per person.

**Figure 1:**
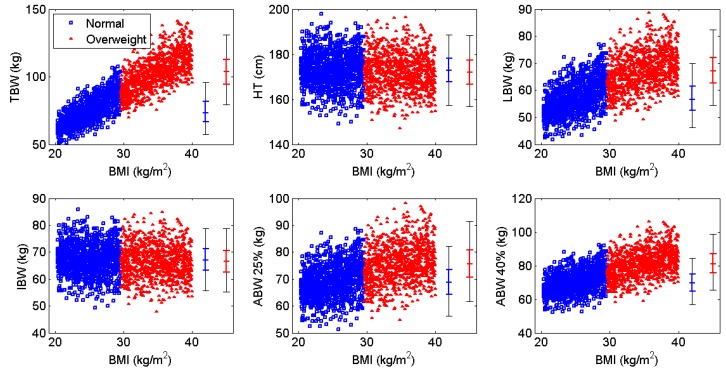
Correlation of body weight metrics with body mass index (BMI) for each BMI subgroup (blue = normal weight, red = overweight and obese). TBW: total body weight; HT: height; LBW: lean body weight; IBW: ideal body weight; ABW: adjusted body weight.

Following this initial investigation, we explored dosing regimens using alternative weight metrics. The correlation between each weight metric and BMI is shown in Figure 1. We began by administering a dose of 10 IU kg^−1^ of each weight metric on a Q48 h dosing schedule. Once steady state was reached, the percentage of patients with C_min_ ≥ 1.0 IU dL^−1^ was calculated. If this percentage was below 95%, the dose was incrementally increased until this threshold was reached. We then calculated the mean weekly consumption associated with the minimum dose required to reach the safety criterion for each metric to assess cost-effectiveness. Since a Monday-Wednesday-Friday dosing schedule is commonly used in hemophilia A prophylaxis, we performed analogous simulations using this schedule instead of a regular 48 h interval. We used the optimal doses found in the previous study on Monday and Wednesday, and then increased the dose on Fridays to compensate for the longer interval until the safety criterion was met.

**Figure 2:**
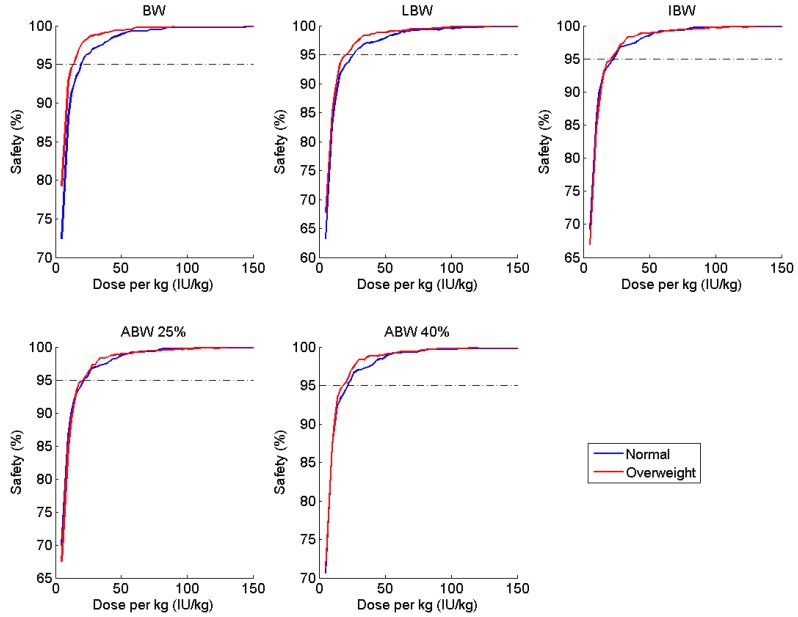
Percentage of patients with C_min_ ≥ 1 IU dL^−1^ (safety) at various doses per kg of various weight metrics, stratified by BMI subgroup, administered at 48-h intervals.

**Figure 3:**
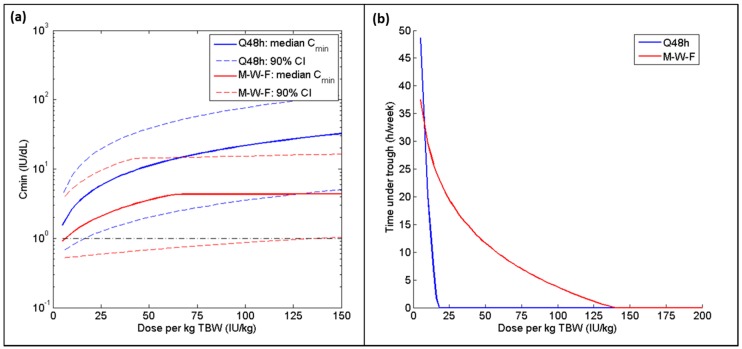
(**a**) Median and 90% confidence intervals for C_min_ and (**b**) 95th quantile for time spent below 1 IU dL^−1^ (hours per week) for TBW-based dosing regimen administered at different intervals for the combined group (normal + overweight/obese) for both Q48 h (blue) and Monday-Wednesday-Friday (red) dosing schedules. For the Q48 h regimen, all doses are increasing along the *X*-axis; for the Monday-Wednesday-Friday schedule, only the Friday dose is changing (Monday and Wednesday doses are fixed at 20 IU per kg TBW).

Table 3 and Table 4 summarize the doses per kg of each weight metric required to reach the 95% safety criterion (when infused every 48 h or Monday-Wednesday-Friday, respectively) and the associated weekly consumption in each of the BMI categories and in the merged population, assuming a baseline factor level of 0.5 IU dL^−1^. The most appropriate regimen is the one that meets the safety requirements while consuming the least amount of factor concentrate. For patients within the normal BMI range, LBW produced the optimal regimen for both dosing schedules; for the overweight and obese cohort, an IBW-based dosing regimen was found to be most cost-effective. Furthermore, the range of mean weekly consumption across the various weight metrics was much tighter for the normal BMI subgroup (125 IU per person per week) as compared to the overweight/obese subgroup (483 IU per person per week). When the two subgroups were combined, ABW with a 25% correction factor proved to be ideal for the Q48 h regimen, with IBW a very close second with a difference of just 5 IU per person per week. Both ABW_25_ and IBW perform almost identically in terms of safety for both BMI subgroups for the Q48 h regimen (Figure 2). However, IBW performed better than all other weight metrics when a Monday-Wednesday-Friday schedule was adopted, with a difference in consumption of over 100 IU per person per week when compared to the next best metric (LBW). Nevertheless, the amount of time spent below 1 IU dL^−1^ is significantly greater when following a Monday-Wednesday-Friday regimen as compared to the Q48 h dosing schedule (Figure 3b); additionally, an extremely high Friday dose (>125 IU kg^−1^ TBW) is required to meet the 95% safety requirement, whereas a dose of 18 IU kg^−1^ TBW is successful for the Q48 h regimen (Figure 3a).

**Figure 4:**
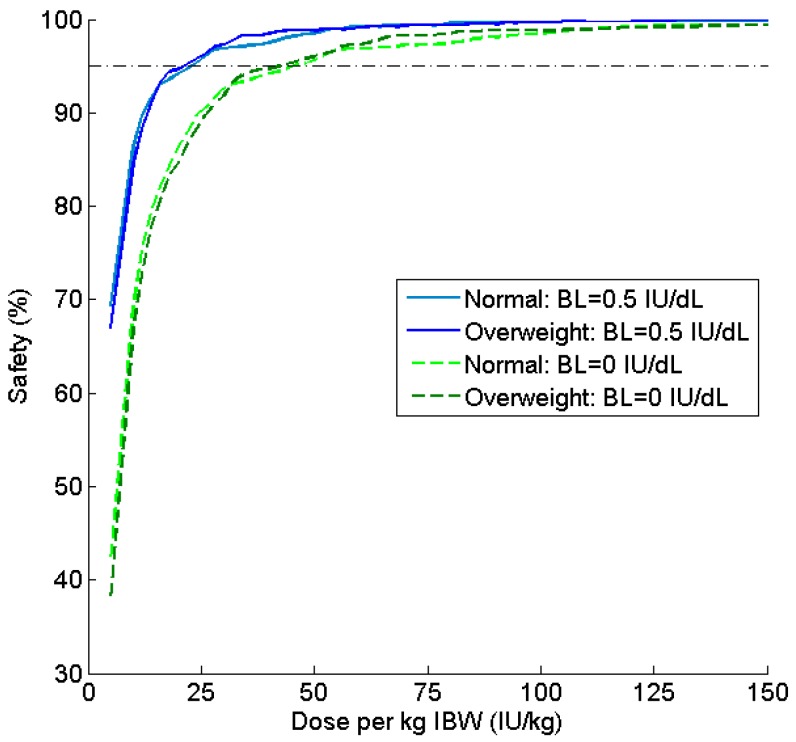
Comparison of safety profiles for patients simulated with baseline 0.5 IU dL^−1^ and 0 IU dL^−1^ for a Q48 h regimen. Safety (%) is the percentage of patients with C_min_ ≥ 1 IU dL^−1^ at various doses per kg of IBW.

Ideal body weight continued to perform well in simulations with an assumed baseline of 0 IU dL^−1^. The safety ratio versus dose curves are once again nearly identical for both BMI subgroups (Figure 4), although consumption was approximately doubled as compared to the Q48 h regimen.

## 4. Discussion

We began by assessing the safety and cost-effectiveness of a typical 20 IU kg^−1^ TBW, Q48 h regimen in an overweight and obese patient population. For comparison, we determined the TBW-based dose required to meet the safety criterion. At a dose of 14 IU kg^−1^ TBW, 95% of patients had FVIII levels of at least 1 IU dL^−1^ at all times; the median C_min_ was 3.9 IU dL^−1^ and the mean consumption was just over 5000 IU per person per week. By contrast, the 20 IU kg^−1^ TBW regimen produced a median C_min_ of 5.4 IU dL^−1^ with a mean consumption of 7250 IU per person per week. Hence, the standard TBW-based dosing protocol results in over 40% higher consumption than required in the overweight and obese population; assuming a cost of $1 US per unit of concentrate, this amounts to over $100,000 US in excess spending per person annually. From this evaluation, it is clear that TBW does not represent the optimal body weight metric to guide FVIII dosing.

Simulations using dosing regimens based on alternative weight metrics (LBW, IBW, ABW_25_, and ABW_40_) were carried out using the two most common dosing schedules in hemophilia A prophylaxis: a regular 48 h regimen and a Monday-Wednesday-Friday regimen. Adapting a Monday-Wednesday-Friday timetable made it extremely difficult to meet the safety requirement, regardless of which weight metric was used to define the dose. While patients are often advised to increase their FVIII dose on Friday, a simple doubling of the dose is not sufficient. A potentially harmful Friday dose of 140 IU kg^−1^ TBW was required for 95% of patients to have a C_min_ ≥ 1 IU dL^−1^, compared to 18 IU kg^−1^ TBW to meet this safety minimum when infused every 48 h. Furthermore, the time spent below 1 IU dL^−1^ (and, consequently, the risk of bleeding events [32]) is significantly greater when following a Monday-Wednesday-Friday regimen, even if the Friday dose is twice or three times greater than the Monday and Wednesday doses (Figure 3b). In fact, a 2010 study in which FVIII was administered three times per week found that over 80% of bleeds occurred 48–72 h post-infusion [33]. The Monday-Wednesday-Friday treatment schedule, while more convenient, is no longer considered to be optimal therapy due to this increased vulnerability to bleeds during the weekend, with alternate day dosing representing the ideal regimen [34,35].

Due to analytical limitations, it can be difficult to obtain an exact measure of a patient’s baseline factor level. Many assays have a lower limit of quantification of 1 IU dL^−1^ [36,37], which is greater than endogenous levels for severe hemophilia patients. To balance both safety and resource utilization, we ran initial simulations with an assumed baseline of 0.5 IU dL^−1^. However, it is known that many severe hemophilia patients possess a genetic mutation such that no functional FVIII is produced endogenously. For this reason, the simulations were performed again using a baseline of 0 IU dL^−1^ to ensure similar trends were observed within this sub-population. Notably, a 95% safe ratio can be achieved in a population with no endogenous FVIII production at a reasonable dose (34 IU kg^−1^ TBW) if administered every 48 h. However, it is not possible to meet that safety threshold in this population if a Monday-Wednesday-Friday dosing schedule is employed. If the safety criteria is lowered to 90%, it can be met, but only with extremely high Friday doses (between 130 and 180 IU kg^−1^ for the various weight metrics) and associated weekly consumption (>16,000 IU per person per week); a study by Collins et al. found similarly high doses (>100 IU kg^−1^ for patients with average half-lives, and up to 400 IU kg^−1^ in extreme cases) were required to maintain FVIII levels above 1 IU dL^−1^ throughout the week when following this dosing schedule [38]. These results suggest that a regular dosing interval of 48 h offers significant advantages over the weekly Monday-Wednesday-Friday schedule in terms of both safety and cost-effectiveness.

After exploring all combinations of dosing schedule and baseline factor level, we determined that IBW-based dosing provides a safe and cost-effective regimen in the majority of scenarios, with ABW_25_ producing fairly similar results. Ideal body weight performed almost exactly the same in terms of safety between the normal and overweight groups across all of the doses and regardless of baseline, as evidenced by the closeness of the curves shown in Figure 2 and Figure 4. Further, IBW was the most cost-effective in three out of four simulations; in the fourth, it differed by only 5 IU per person per week from the optimal regimen (ABW_25_). If we compare the optimal regimen for a Q48 h schedule with a baseline of 0.5 IU dL^−1^ (i.e., 20.7 IU kg^−1^ IBW) to a 20 IU kg^−1^ TBW, this alterative regimen offers a savings of over 2000 IU per person per week (or nearly $110,000 US annually) for overweight and obese patients. Thus, IBW-based dosing offers a similar safety profile to the currently used TBW strategy while moderating the economic burden of clotting factor prophylaxis.

This exercise was limited by the constraints of the data. The model used herein was built on PK data from a specific brand of FVIII concentrate, although brand has not generally been found to significantly influence PK. A second limitation to the applicability of this approach is that the source data is largely from older (10+ years of age) patients, and the opinions on use of prophylaxis in adults are varied [39,40,41]. Obesity rates are also increasing rapidly amongst pediatric patients and similar dosing adjustments are likely appropriate in this population, but cannot be confirmed in this study. Further study of pediatric populations (and validated pediatric population PK models) is required in order to determine a dosing regimen that applies not only to all BMI’s but also to all ages.

As the prevalence of obesity has risen in the general population, a number of studies have been conducted to investigate the frequency of overweight and obesity among hemophilia patients, complications such as co-morbidities and decreased quality of life, and recommendations for management strategies. Many pharmacokinetic studies exploring the relationship between excess body weight and plasma volume (and, by extension, in vivo recovery) have postulated that dosing according to body weight results in overdosing and an ineffective use of resources, suggesting instead that dosing be guided by LBW or IBW [42,43]. This study compared several weight metrics and confirmed that an IBW-based regimen is both safe and cost-effective across a range of BMI’s. Ideal body weight produced slightly better results than other weight metrics because it is calculated based solely on height; as shown in Figure 1, there is no correlation between IBW and BMI as observed with the other metrics investigated.

Although we were able to identify a weight metric that is more suitable for a variable population, the high inter-individual variability in PK handling of factor concentrates precludes the definition of a single, “one dose fits all” strategy. In order to optimize prophylaxis, regimens should be tailored to the individual PK profile. This process has been facilitated by the development of the WAPPS-Hemo service (www.wapps-hemo.org), a Canadian-based user-friendly and industry-independent platform that produces estimates of individual PK parameters through a Bayesian iterative approach. The WAPPS-Hemo service also includes a module for dosing regimen development, wherein clinicians can predict the effects of changing dose, frequency, or targeted trough for a specific patient before implementing these changes in practice. While PK-tailored dosing regimens may offer the best results, weight-based strategies are still the norm, but these can be optimized by adapting a different weight metric (i.e., IBW) to guide safe and cost-effective dosing at a population level.

## 5. Conclusions

In summary, we conducted simulations based on a previously published model of a conventional FVIII to explore the appropriateness of different weight metric-based dosing regimens for hemophilia A prophylaxis for overweight and obese patients. Regimens were required to produce a C_min_ ≥ 1 IU dL^−1^ in 95% of the population, and then the average consumption for each regimen was calculated to evaluate resource-effectiveness. From this study, we conclude that ideal body weight performs the best, maintaining safety while tempering factor consumption for overweight and obese patients.

## Figures and Tables

**Table 1:** Details of the model developed by Garmann et al. [31]. CL: clearance; Q: intercompartmental clearance; V_1_: volume of the central compartment; V_2_: volume of the peripheral compartment; RUV: residual unexplained variability; BSV: between subject variability; LBW: lean body weight.

Parameter	Estimate	Covariate Effect	BSV (%CV)
CL (dL∙h^−1^)	1.88	$\theta_{\mathrm{CL}}{\left(\frac{\mathrm{LBW}}{51.1}\right)}^{0.610}$	37.0
Q (dL∙h^−1^)	1.90		
V_1_ (dL)	30.0	$\theta_{V_{1}}{\left(\frac{\mathrm{LBW}}{51.1}\right)}^{0.950}$	11.2
V_2_ (dL)	6.37		
Proportional RUV (%CV)	26.7		
Additive RUV (IU dL^−1^)	1.10		

**Table 2:** Comparison of the typical 20 IU kg^−1^ total body weight (TBW) dose and the lowest dose meeting the safety threshold (i.e., 14 IU kg^−1^ TBW) in overweight and obese patients. Results are presented as median (90% confidence interval).

Measure	Regimen	
	20 IU kg^−1^ TBW, Q48 h	14 IU kg^−1^ TBW, Q48 h
**C_min_ (IU dL^−1^)**	5.4 (1.2–17.3)	3.9 (1.0–12.3)
**Consumption (IU per person per week)**	7260 (5730–8780)	5080 (4010–6140)

**Table 3:** Summary of safety and economic evaluations of different weight metrics used in a Q48 h regimen across BMI subgroups, assuming a baseline factor level of 0.5 IU dL^−1^. Dose is the dose required to have 95% of patients with a steady state C_min_ over 1 IU dL^−1^. Optimal regimens for each subgroup and the overall population are bolded. IBW: ideal body weight, ABW: adjusted body weight.

Metric	Normal		Overweight and Obese		All BMI Categories		
	Dose (IU kg^−1^)	Mean Consumption (IU per Person per Week)	Dose (IU kg^−1^)	Consumption (IU per Person per Week)	Dose (IU kg^−1^)	Mean Consumption (IU per Person per Week)	Difference in Consumption from TBW
TBW	20.0	5202	14.0	5074	18.0	5603	-
LBW	**25.6**	**5114**	21.3	5028	23.8	5186	−417
IBW	22.2	5222	**20.7**	**4828**	22.1	5176	−427
ABW_25_	21.7	5239	20.0	5311	**20.4**	**5171**	**−432**
ABW_40_	21.1	5173	18.0	5129	20.0	5301	−302

**Table 4:** Summary of safety and economic evaluations of different weight metrics used in a Monday-Wednesday-Friday regimen across BMI subgroups, assuming a baseline factor level of 0.5 IU dL^−1^. Dose is the Friday dose required to have 90% of patients with a weekly C_min_ ≥ 1 IU dL^−1^. Optimal regimens for each subgroup and the overall population are bolded.

Metric	Normal		Overweight and Obese		All BMI Categories		
	Dose (IU kg^−1^)	Consumption (IU per Person per Week)	Dose (IU kg^−1^)	Consumption (IU per Person per Week)	Dose (IU kg^−1^)	Consumption (IU per Person per Week)	Difference in Consumption from TBW
TBW	74	8174	54	9320	62	8716	-
LBW	**94**	**8082**	82	8740	88	8442	−274
IBW	78	8213	**84**	**8543**	**80**	**8312**	**−404**
ABW_25_	78	8195	72	8558	76	8459	−258
ABW_40_	76	8126	68	8792	72	8481	−235

## Supplementary Materials

The following are available online at www.mdpi.com/1999-4923/9/4/47/s1, Figure S1: Correlation plots for all body size metrics used in simulations for the normal BMI subgroup. Diagonal elements contain histograms. Figure S2: Correlation plots for all body size metrics used in simulations for the overweight/obese subgroup. Diagonal elements contain histograms.
