# Supplementary material for: Modeling of Body Weight Metrics for Effective and Cost-Efficient Conventional Factor VIII Dosing in Hemophilia A Prophylaxis
Source: Pharmaceutics. 2017 Oct 17;9(4):47. doi: 10.3390/pharmaceutics9040047 (PMC5750653; doi:10.3390/pharmaceutics9040047)
Supplement: Supplementary file 1 [file pharmaceutics-09-00047-s001.pdf]

Supplementary Materials: Modeling of Body Weight Metrics for Effective and Cost-Efficient Conventional Factor VIII Dosing in Hemophilia A Prophylaxis

Alanna McEneny-King [1], Pierre Chelle [1], Severine Henrard [2], Cedric Hermans [3], Alfonso Iorio [4,5] and Andrea N. Edginton [1,*]

[1] School of Pharmacy, University of Waterloo, Waterloo, Ontario, Canada
[2] Louvain Drug Research Institute, Clinical Pharmacy Research Group and Institute of Health and Society (IRSS), Université catholique de Louvain, Brussels, Belgium
[3] Haemostasis and Thrombosis Unit, Division of Haematology, Cliniques universitaires Saint-Luc, Université catholique de Louvain, Brussels, Belgium
[4] Department of Health Evidence, Research Methods and Impact, McMaster University, Hamilton, Ontario, Canada
[5] Department of Medicine, McMaster University, Canada
* Correspondence: aedginto@uwaterloo.ca; Tel.: 519-888-4567 (ext. 21315)

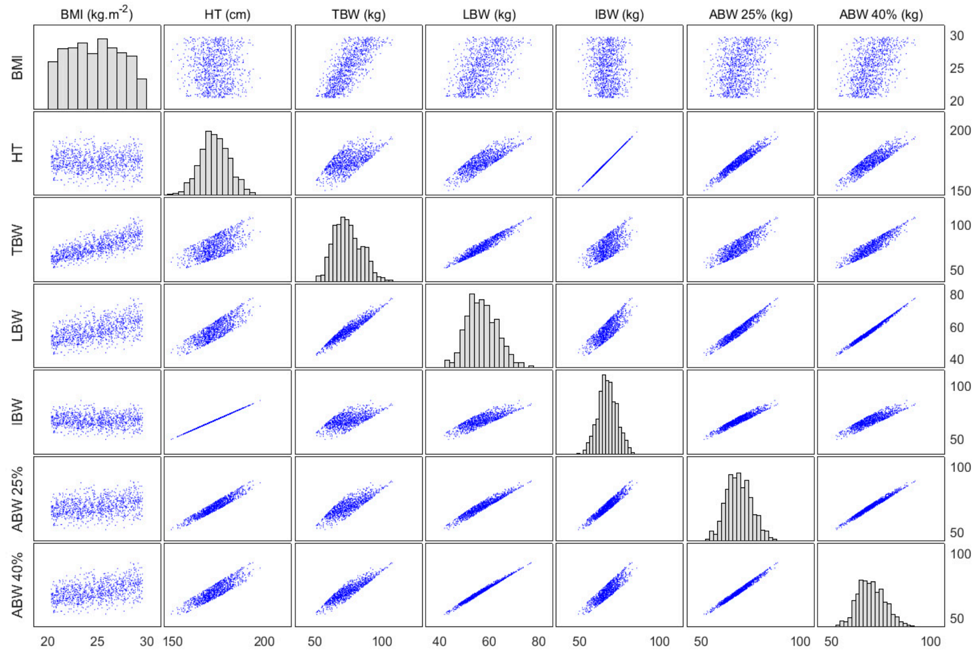

Figure S1. Correlation plots for all body size metrics used in simulations for the normal BMI subgroup. Diagonal elements contain histograms.

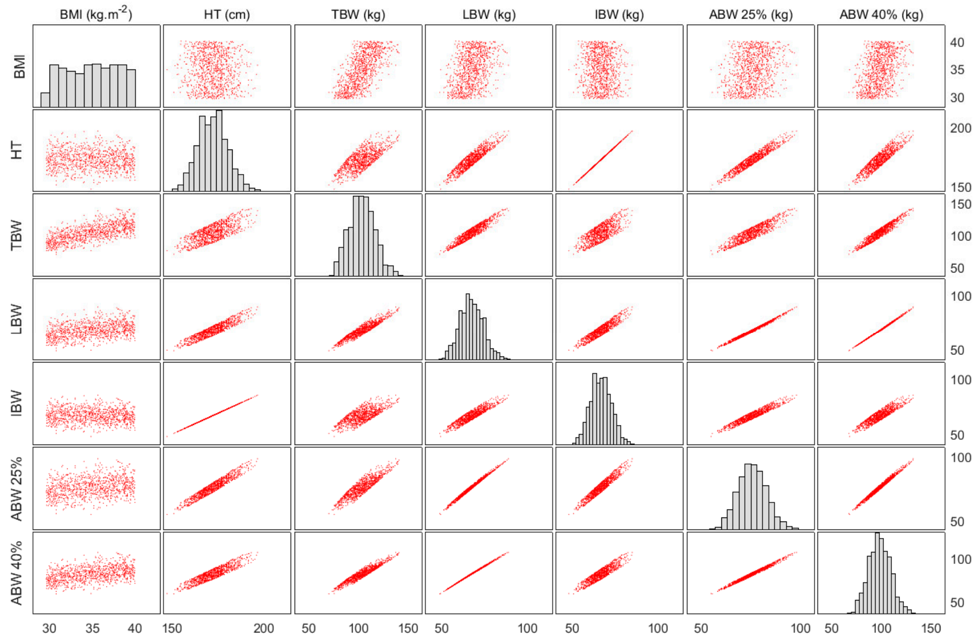

Figure S2. Correlation plots for all body size metrics used in simulations for the overweight/obese subgroup. Diagonal elements contain histograms.
